# Methylene blue removal from water using chaya-derived biochar: Effect of metabolite extraction on adsorption

**DOI:** 10.1371/journal.pone.0358748

**Published:** 2026-09-24

**Authors:** Viviana Roche-Llerena, Leonardo Hernández, Raúl Pareja-Rodríguez, Geonel Rodríguez-Gattorno, María A. Fernández-Herrera

**Affiliations:** Departamento de Física Aplicada, Centro de Investigación y de Estudios Avanzados del Instituto Politécnico Nacional, Unidad Mérida, Mérida, Yucatán, Mexico; Konkuk University, KOREA, REPUBLIC OF

## Abstract

This study evaluated biochars derived from *Cnidoscolus aconitifolius* (chaya) leaf residues for methylene blue (MB) removal from water. Two materials were produced under identical thermal conditions from untreated biomass (CCP-A) and post-extraction biomass (CCP-B), allowing direct assessment of the effect of metabolite extraction. Characterization by SEM/EDS, XRD, FTIR, Raman spectroscopy, XPS, and N_2_ adsorption-desorption showed that extraction altered surface chemistry and textural properties, increasing oxygen-containing functionalities and BET surface area from 1.92 to 6.10 m^2^ g^-1^ and decreasing mean pore diameter from 14.1 to 8.4 nm. These differences were associated with improved MB removal by CCP-B under dark and irradiation conditions. Kinetic analysis was consistent with multiple transport contributions. Among the equilibrium models, Langmuir yielded the highest linearized R^2^ and a model-estimated q_max_ of 155.3 mg g^-1^, whereas degrees-of-freedom-adjusted RMSE analysis on the original q_e_ scale did not identify a single unequivocally superior model. Despite its relatively low BET surface area, the MB uptake of CCP-B suggests that adsorption cannot be explained solely by the dry surface accessible to N_2_ at 77 K. Oxygen-containing functionalities may contribute to electrostatic attraction and hydrogen bonding, whereas aromatic domains may support π-π interactions. Under UV and simulated solar irradiation, CCP-B reached overall removal efficiencies of 98.6% and 93.8%, respectively. Because adsorption and possible light-induced transformations were not quantified independently, these values represent overall photo-assisted removal. Inhibition by isopropanol was consistent with possible participation of hydroxyl radicals or related reactive oxygen species; however, this evidence was indirect, the species were not detected directly, and degradation products and mineralization were not evaluated. CCP-B removal efficiency decreased from approximately 92% to 31% over five adsorption-drying reuse cycles, indicating limited reuse performance without effective regeneration. Under the conditions evaluated, metabolite extraction before carbonization modified the surface properties of chaya-derived biochar and was associated with improved MB removal.

## Introduction

The discharge of dye-containing wastewater remains a significant environmental challenge due to the persistence and toxicity of many synthetic colorants [1,2]. Among them, methylene blue (MB) is widely used in textile, paper, plastic, and pharmaceutical industries and is frequently employed as a model contaminant for evaluating water treatment technologies [3]. The presence of MB in aquatic systems can reduce light penetration, affect photosynthetic processes, and generate adverse effects on aquatic organisms, highlighting the need for efficient and sustainable removal strategies [4].

Adsorption is one of the most widely applied approaches for dye removal because of its operational simplicity, relatively low cost, and high efficiency [5]. In recent years, biomass-derived biochars have attracted considerable attention as adsorbents due to their renewable origin and tunable physicochemical properties [6–8]. Their performance is commonly associated with surface area, pore structure, mineral composition, and the presence of oxygen-containing functional groups capable of interacting with organic pollutants through electrostatic attraction, hydrogen bonding, and π-π interactions [9]. Some biochar-based systems have shown increased MB removal under irradiation through concurrent adsorption and photolysis [1,10,11].

Despite these advances, high adsorption performance in biomass-derived biochars is frequently achieved through chemical activation or post-carbonization functionalization, which introduces additional processing steps and chemical inputs [12]. Biochars prepared from post-extraction plant residues have also been investigated for methylene blue adsorption; however, some of these materials require subsequent chemical activation. More importantly, direct comparisons between biochars produced from the same biomass before and after metabolite extraction, under identical carbonization conditions, remain limited. Such paired comparisons are necessary to isolate the effect of precursor extraction from differences caused by biomass source or processing conditions. Therefore, it remains unclear whether metabolite extraction alone can modify the surface chemistry and adsorption performance of the resulting biochar without additional chemical activation.

Chaya (*Cnidoscolus aconitifolius*) is a perennial shrub native to the Yucatán Peninsula and is widely recognized for its nutritional and medicinal value [13]. Its leaves contain phenolic and terpenoid constituents, and taraxerone, a carbon-rich pentacyclic triterpenoid, has been isolated from isopropanol extracts of chaya leaves [13–17]. These extractable compounds may undergo thermal reactions different from those of the structural biopolymers. Thermolabile constituents may volatilize or undergo fragmentation, whereas aromatic compounds may participate in condensation and char-forming reactions. Their partial removal may therefore alter the relative composition of the precursor and the balance among volatile release, condensation, aromatization, and partial oxidation during heating, thereby affecting pore development, structural disorder, and surface oxygen functionalities. Because isopropanol extraction removes a mixture rather than a single metabolite class, these effects were considered a working hypothesis. We hypothesized that the post-extraction residue would produce a carbonaceous material with different surface chemistry and textural properties, including a higher relative abundance of oxygen-containing groups and greater affinity for methylene blue, than untreated chaya leaves subjected to the same thermal conditions.

In this work, biochars were produced from untreated and post-extraction chaya leaves under the same thermal conditions. The materials were systematically compared using structural, textural, and surface characterization techniques, and their methylene blue removal performance was evaluated under dark and irradiation conditions. The objective was to determine whether metabolite extraction prior to carbonization modifies the physicochemical and adsorption properties of chaya-derived biochar and whether post-extraction biomass can be valorized as an adsorbent without additional chemical activation.

## Materials and methods

### Biomass source and preparation

Chaya leaves (*Cnidoscolus aconitifolius*) were collected in March 2024 from a privately owned property in Dzityá, Mérida, Yucatán, Mexico (21°03′25.2″ N, 89°39′56.8″ W; 10 m above sea level), with permission from the landowner. No governmental collection permit was required because the plant material was collected on private property outside a protected natural area. The plant material was taxonomically identified by Biol. J. L. Tapia-Muñoz at the “U Najil Tikin Xiw” Herbarium of the Centro de Investigación Científica de Yucatán, A.C. (CICY), where a voucher specimen was deposited under voucher number 001 [16]. The post-extraction residue used to produce CCP-B was obtained following the procedure reported by Hernández et al. [17]. Briefly, dried and ground chaya leaves were extracted with isopropanol at a solvent-to-biomass ratio of 10:1 mL g^-1^ for 48 h at 25 °C under ambient conditions. After extraction, the solid residue was recovered by filtration, dried, and stored for subsequent carbonization. The extract yield was approximately 9%. Both the dehydrated leaf powder and the post-extraction residue were stored in sealed glass containers to minimize contamination and moisture absorption.

### Carbonaceous material synthesis

Thermogravimetric analysis (TGA) and derivative thermogravimetry (DTG) of the chaya leaves and post-extraction residue were performed using a TA Instruments Discovery system under flowing air (25 mL min^-1^). Samples were heated from ambient temperature to 600 °C and 800 °C, respectively, at a rate of 10 °C min^-1^. The TGA/DTG profiles are presented in S10 Fig in S1 File and were used to guide the selection of the treatment temperature. The last major DTG maximum of the more thermally resistant post-extraction residue occurred at 416 °C; therefore, 450 °C was selected as an operational temperature above this maximum while avoiding prolonged exposure to higher temperatures and additional oxidative mass loss, consistent with thermal treatments reported for related biomass-derived carbonaceous materials [12,18]. Because TGA/DTG was conducted under flowing air, whereas the furnace treatment was performed under static air, the thermal analysis was used as a selection guide rather than as a direct simulation of the synthesis conditions. Materials prepared at lower or higher temperatures were not compared; therefore, 450 °C is described as selected rather than optimal.

Both precursors were subjected to identical thermal-treatment conditions. The dehydrated leaf powder and post-extraction residue were separately heated in a Thermo Scientific muffle furnace to 450 °C at a rate of 10 °C min^-1^ and maintained at this temperature for 30 min under static air. This controlled oxidative treatment was selected as a simple approach to obtain carbonaceous materials containing oxygen-bearing surface functionalities without subsequent oxidation or chemical activation. Previous studies have shown that oxidative thermal treatment can promote the formation or retention of hydroxyl, carbonyl, and carboxyl functionalities within a carbonaceous framework [12,18]. The resulting materials were designated CCP-A, derived from dehydrated chaya leaf powder, and CCP-B, derived from the post-extraction residue.

### Characterization techniques and equipment

The carbonaceous materials were characterized using several complementary analytical techniques. Scanning electron microscopy coupled with energy-dispersive X-ray spectroscopy (SEM–EDS) was used to examine the surface morphology and elemental composition of the carbonaceous powders before and after exposure to MB solutions. Images were acquired at different magnifications using a JEOL JSM-7600F microscope equipped with a low-angle backscattered-electron (LABE) detector and operated at accelerating voltages of 10−15 kV. X-ray diffraction (XRD) patterns were recorded using a Bruker D8 Advance diffractometer with Cu Kα radiation (λ = 1.5418 Å), operated at 40 kV and 30 mA. Measurements were performed over a 2θ range of 5−60°, with a step size of 0.02° and an exposure time of 0.5 s per step. X-ray photoelectron spectroscopy (XPS) analyses were performed using a Thermo Scientific K-Alpha surface analysis system equipped with a monochromated Al Kα X-ray source (1486.6 eV). The source was operated at 12 kV and 40 W and focused on a nominal 400 µm analysis area, with the X-rays incident at an angle of 30° relative to the sample surface. Survey spectra were acquired over a binding-energy range of 0−1350 eV using an energy step of 1 eV and a pass energy of 100 eV. High-resolution spectra were acquired using an energy step of 0.1 eV and a pass energy of 50 eV. Binding energies were calibrated against the adventitious C 1s peak at 284.5 eV, and spectral deconvolution was performed using OriginPro 2021. Raman spectra were acquired using a WITec ALPHA 300RA confocal micro-Raman spectrometer with a 488 nm laser operated at 2.8 µW to minimize sample damage. Five randomly selected points were analyzed for each sample, and the averaged spectrum is reported. Fourier-transform infrared (FTIR) spectra of CCP-A and CCP-B were recorded using an Agilent Cary 630 FTIR spectrometer equipped with an attenuated total reflectance (ATR) accessory. Spectra were collected over the range of 4000−600 cm^-1^ using 32 scans per spectrum and are presented in S9 Fig in S1 File.

### Methylene blue removal experiments

Methylene blue (MB) removal was evaluated using three complementary experimental protocols: adsorption under dark conditions, sequential dark adsorption followed by irradiation, and direct simulated solar irradiation. In all experiments, the amount of 6 mg of CCP-A or CCP-B was added to 30 mL of an aqueous MB solution with an initial concentration of 29 mg/L (Sigma-Aldrich, 99%), and the suspensions were continuously stirred at 300 rpm. For the dark adsorption experiments, the biochar-MB suspensions were maintained in the dark for 24 h. For the sequential dark adsorption-irradiation experiments, the suspensions were first maintained under dark conditions for 24 h and subsequently exposed either to UV irradiation at 365 nm (approximately 0.06 W/m^2^, 8 cm from the light source) for an additional 24 h or to simulated solar irradiation provided by a xenon lamp (1000 W/m^2^, 12 cm from the light source) for an additional 5 h. The final MB concentrations obtained after the complete sequential treatments are presented in Fig 3 and Table 3.

In a separate direct-irradiation experiment, MB concentration was monitored during 5 h of simulated solar irradiation in the presence of CCP-A or CCP-B, without the preceding 24-h dark adsorption stage. These kinetic results are presented in Fig 7. Therefore, the residual MB concentrations reported in Fig 7 and Table 3 were obtained using different experimental protocols and should not be interpreted as replicate measurements of the same experiment. MB concentrations were determined by UV-Vis spectroscopy at 664 nm, the absorption maximum of MB, using an Agilent spectrophotometer and a previously established calibration curve (S1 Fig in S1 File). At each sampling point, a 1 mL aliquot was withdrawn and centrifuged at 4,500 rpm for 10 min to separate the suspended carbonaceous particles. The absorbance of the resulting supernatant was then measured at 664 nm using deionized water as the blank. After measurement, each analyzed aliquot was returned to its corresponding experimental suspension to maintain a constant working volume throughout the experiment. All experiments were performed in triplicate, and the reported values represent the means of three independent experiments. The corresponding control experiments are presented in S2 Fig in S1 File. The decrease in MB concentration under dark conditions was attributed to adsorption. In contrast, the decrease observed during or after irradiation was reported as overall photo-assisted removal because adsorption and possible light-induced transformations may occur concurrently and their individual contributions were not quantified independently. Accordingly, the irradiated experiments were not interpreted as direct evidence of MB photodegradation.

### Kinetic and equilibrium modeling

Adsorption kinetics were analyzed using pseudo-first-order, pseudo-second-order, intraparticle diffusion, and Elovich models. Equilibrium data were fitted to Langmuir, Freundlich, and Temkin isotherms.

Model parameters were obtained from the corresponding regression equations. Goodness of fit was evaluated using the coefficient of determination (R^2^) and root mean square error adjusted for degrees of freedom (adjusted RMSE), calculated on the original adsorption-capacity scale as:


$$\begin{array}{c} \text{RMSE}=\sqrt{\frac{\sum_{i=\text{1}}^{N}(q_{i,\text{exp}}-q_{i,\text{cal}}{)}^{\text{2}}}{N-p}} \end{array}$$ (1)

where $q_{i,\text{exp}}$ and $q_{i,\text{cal}}$ are the experimental and calculated adsorption capacities ($q_{t}$ for kinetic models and $q_{e}$ for isotherm models), N is the number of observations, and p is the number of fitted parameters. Higher R^2^ and lower adjusted RMSE values indicate better agreement between experimental and calculated adsorption capacities.

## Results and discussion

### Characterizations of biochar

The study first addressed the critical question of how metabolite extraction affects the properties of biomass-derived carbon by comparing materials from native chaya (CCP-A) and post-extraction residue (CCP-B) [16]. The precursor TGA/DTG profiles differed substantially (S10 Fig in S1 File). Dehydrated chaya leaves underwent a dominant mass loss of 84.48% below approximately 200 °C, followed by slower oxidative mass loss. The post-extraction residue showed an initial mass loss of 7.14% and two subsequent mass-loss regions of 49.90% and 31.80%, with DTG maxima at 54, 263, and 416 °C. Above the final major DTG maximum at 416 °C, the mass-loss rate decreased markedly and approached baseline near 500 °C. Thus, 450 °C was selected as an operational temperature above the last major DTG maximum of the more thermally resistant precursor while limiting further oxidative mass loss. These profiles support temperature selection but do not demonstrate that 450 °C is optimal. SEM analysis (see Table 1) revealed distinct morphological differences: CCP-A showed laminar structures approximately 200 nm thick with scaly features, composed mainly of carbon and oxygen with trace potassium, while CCP-B exhibited a cracked surface with potassium-rich agglomerations containing sodium, magnesium, and calcium oxides. Textural characterization by N_2_ adsorption-desorption and BET analysis (S3 Fig in S1 File) showed that CCP-B had a higher $S_{\text{BET}}$ (6.0 vs 1.92 m^2^ g^-1^) and total pore volume (1.3 × 10^−2^ vs 6.8 × 10^−3^ cm^3^ g^-1^), but a lower mean pore diameter (8.4 vs 14.1 nm), than CCP-A. These textural differences are consistent with the distinct morphology observed for CCP-B by SEM. Additional SEM images and EDS elemental maps are presented in S8 Fig in S1 File. Both materials displayed mesoporous characteristics (pore diameters >2 nm), consistent with other biomass-derived carbons reported in the literature [19,20]. The structural modifications induced by metabolite extraction, particularly the increased surface area and reduced pore dimensions, suggest that these materials may exhibit altered adsorption behavior, underscoring the importance of pretreatment strategies for tailoring carbon materials to specific applications.

**Table 1:** Structural and textural properties of chaya-derived carbon materials. SEM images, EDS elemental composition, BET surface area, mean pore diameter, and total pore volume of CCP-A (native biomass) and CCP-B (post-extraction residue).

Material	SEM	EDS		BET analysis	
CCP-A a)	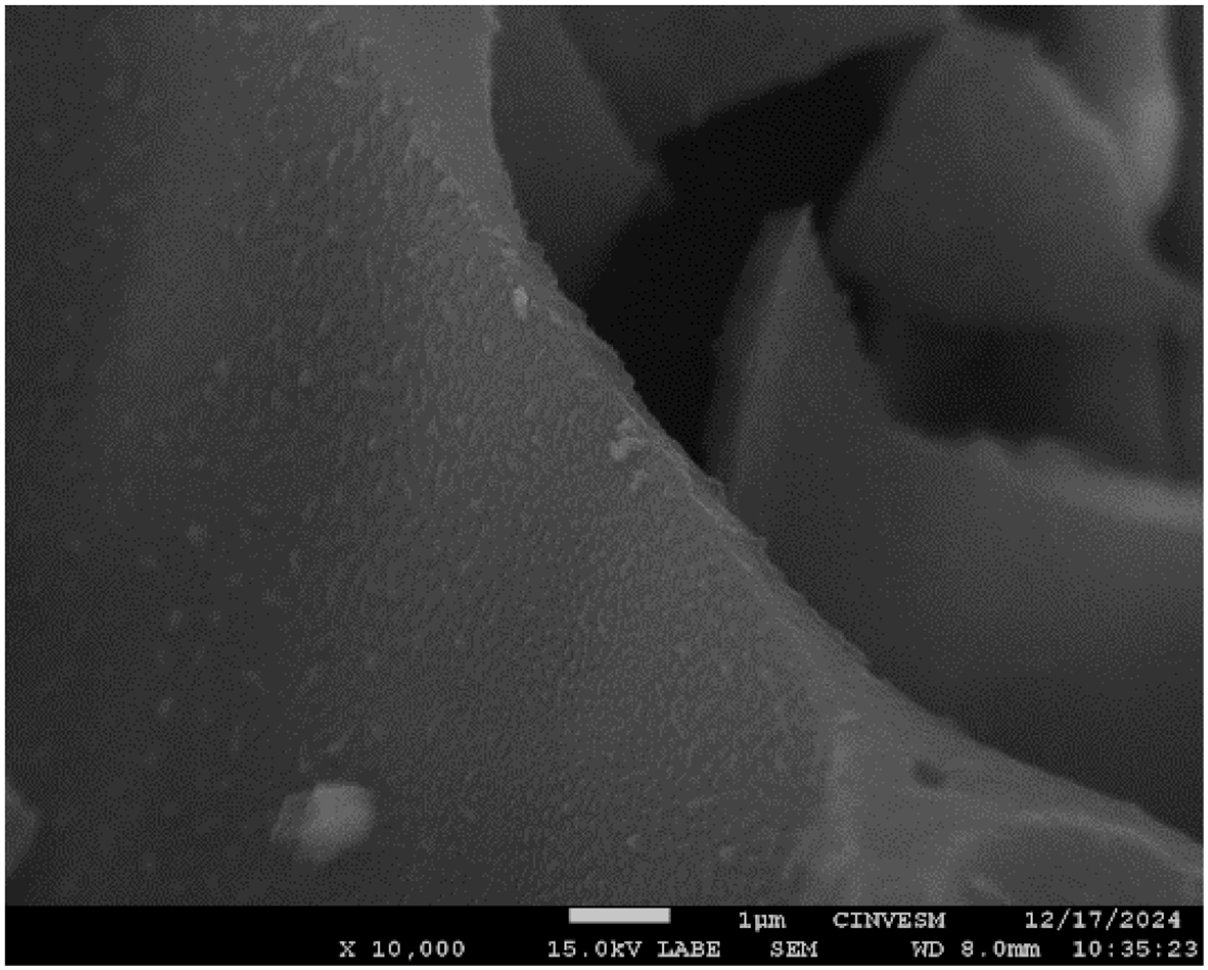	**Element**	**Weight (%)**	Vp (cm^3^ g^-1^)	0.0068
		C	68.4		
		O	21.6	M.P.D (nm)	14.1
		K	5.0		
		Other	5.0	a_s,BET_ (m^2^ g^-1^)	1.92
		Total	100		
CCP-B b)	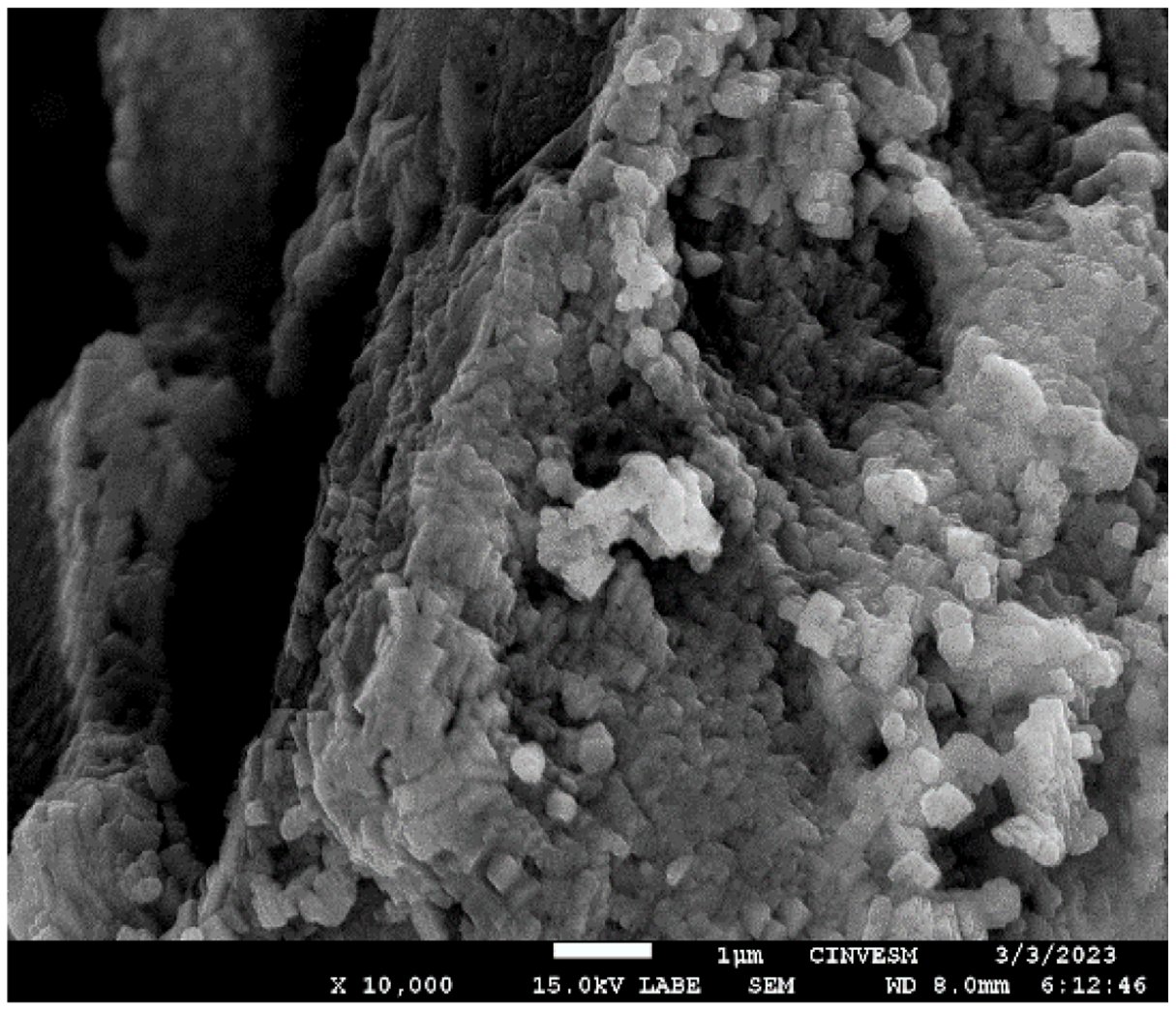	Element	Weight (%)	Vp (cm^3^ g^-1^)	0.013
		C	56.1		
		O	29.2	M.P.D (nm)	8.4
		K	4.7		
		Other	10	a_s,BET_ (m^2^ g^-1^)	6.0
		Total	100		

**Fig 1:**
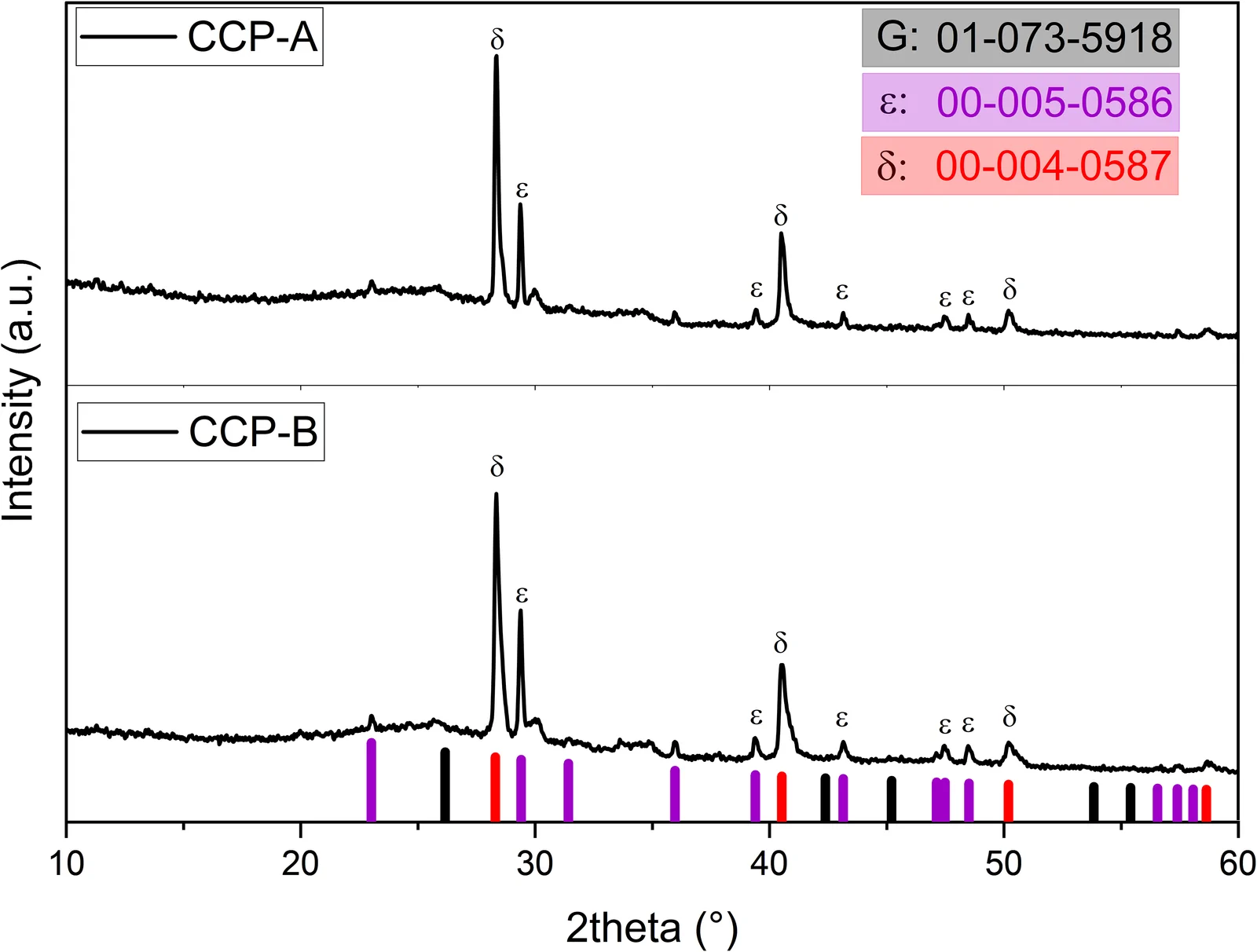
XRD patterns of chaya-derived carbon materials before methylene blue exposure. X-ray diffraction patterns of CCP-A (native biomass) and CCP-B (post-extraction residue). Identified crystalline phases include KCl (δ, PDF #00-004-0587) and CaCO_3_ (ε, PDF #00-005-0586). The reference pattern labeled G corresponds to rhombohedral graphite (PDF #01-073-5918). The broad feature near 2θ=25° is consistent with poorly ordered graphitic carbon.

Metabolite extraction was associated with higher surface oxygen content and differences in the textural and structural properties of CCP-B. A plausible explanation is that removing an isopropanol-soluble fraction containing taraxerone [16] and other extractable constituents changed the relative composition of the precursor and, consequently, the balance among volatile release, condensation, and partial oxidation during heating in air [18]. However, the complete composition of the extract, the elemental composition of the precursors, and the evolved thermal products were not determined. Therefore, the observed differences cannot be assigned to the removal of a specific metabolite or to a confirmed thermal pathway and are interpreted as the net effect of the extraction pretreatment under the conditions evaluated. Because no inert-atmosphere control was included, the contribution of static-air treatment to the resulting surface chemistry and structural disorder cannot be isolated; therefore, the present comparison is limited to the two precursors treated under identical oxidative conditions. XRD analysis of the materials before MB exposure (Fig 1) identified CaCO_3_ and KCl as crystalline mineral phases. CCP-A and CCP-B exhibited reflections at 2θ = 29.4° and 28.4°, assigned to CaCO_3_ (PDF # 00-005-0586) and KCl (PDF # 00-004-0587), respectively, together with a broad feature centered at approximately 2θ = 25°, consistent with poorly ordered carbonaceous domains (PDF # 01-073-5918; ICDD PDF-4 database, Release 2024).

**Fig 2:**
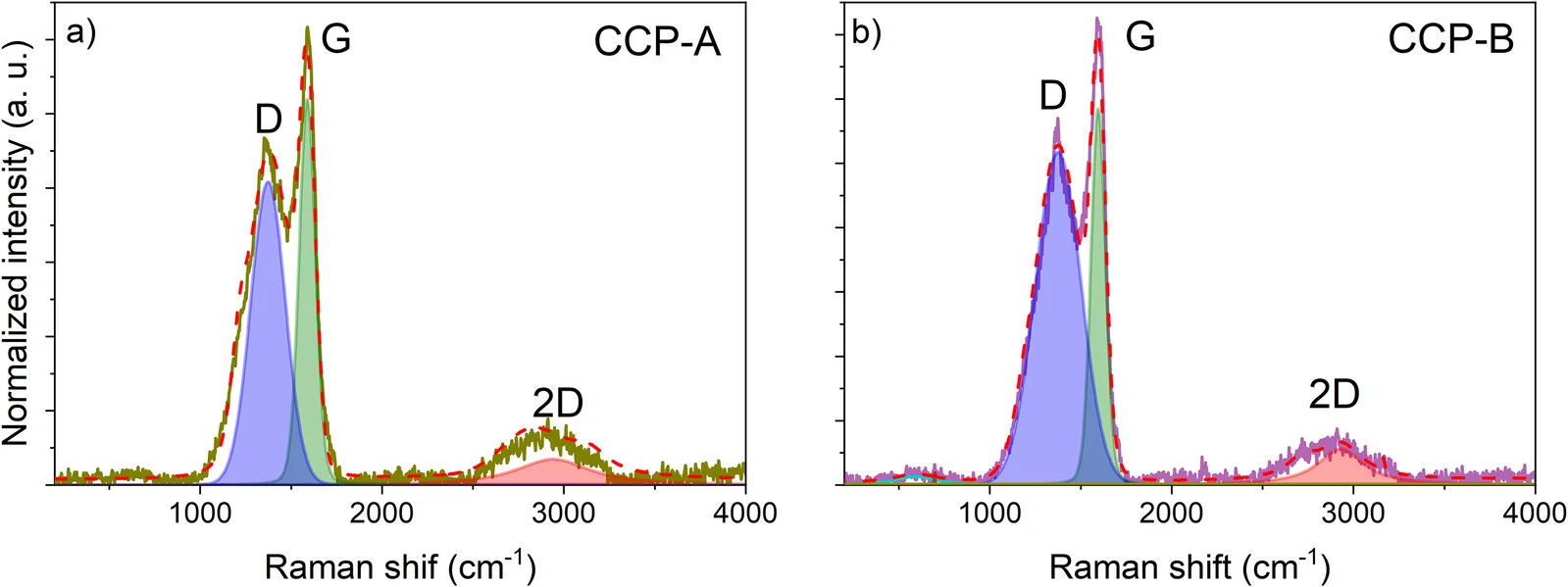
Raman spectra of chaya-derived carbon materials before methylene blue exposure. (a) CCP-A (native biomass) and (b) CCP-B (post-extraction residue).


Following morphological characterization, Raman spectroscopy was used to evaluate the structural organization of CCP-A and CCP-B. The spectra (Fig 2) showed the D and G bands commonly observed in disordered carbon materials [12,18]. The D band at approximately 1360 cm^-1^ is associated with structural disorder and defects, whereas the G band at approximately 1600 cm^-1^ arises from sp^2^-hybridized carbon domains. A broad second-order Raman feature centered at approximately 2860 cm^-1^, conventionally termed the 2D or G’ band, was also observed. This designation is used only as conventional Raman-band nomenclature and is not interpreted as evidence of a two-dimensional material architecture. The *A*_*D*_*/A*_*G*_ ratio was used to compare the relative degree of disorder in the two carbonaceous materials. The average in-plane crystallite size (*L*_*a*_) was estimated using Eq 2:

$$\begin{array}{c} L_{a}=\text{2}.\text{4}\cdot \text{1}{\text{0}}^{\text{-10}}\lambda^{\text{4}}{\left(\frac{A_{D}}{A_{G}}\right)}^{-\text{1}} \end{array}$$ (2)

where λ is the laser wavelength used (488 nm), and *A*_*D*_*/A*_*G*_ is the ratio determined for each material. The *A*_*D*_*/A*_*G*_ and *L*_*a*_ values for both biochars are presented in Table 2.

**Table 2:** Raman-derived structural parameters of chaya-derived carbon materials. *A*_*D*_*/A*_*G*_ ratio and crystallite size (*L*_*a*_) values for CCP-A and CCP-B.

Sample	*A*_*D*_ */A*_*G*_	*L*_*a*_ *(nm)*
CCP-A	1.35	9.41
CCP-B	1.57	8.09

The Raman spectra indicate that both materials contain disordered carbon domains with different degrees of structural organization. CCP-A exhibited a lower *A*_*D*_*/A*_*G*_ ratio and a larger calculated *L*_*a*_ value than CCP-B, indicating a relatively greater degree of structural order. The calculated *L*_*a*_ values are used only as comparative descriptors of local structural organization and not as estimates of particle or sheet dimensions. Conversely, the higher *A*_*D*_*/A*_*G*_ ratio of CCP-B is consistent with a greater density of structural defects. The possible contribution of oxygen-containing functionalities to MB adsorption is evaluated below using the XPS and FTIR results.

### Methylene blue removal under dark and sequential irradiation conditions

**Fig 3:**
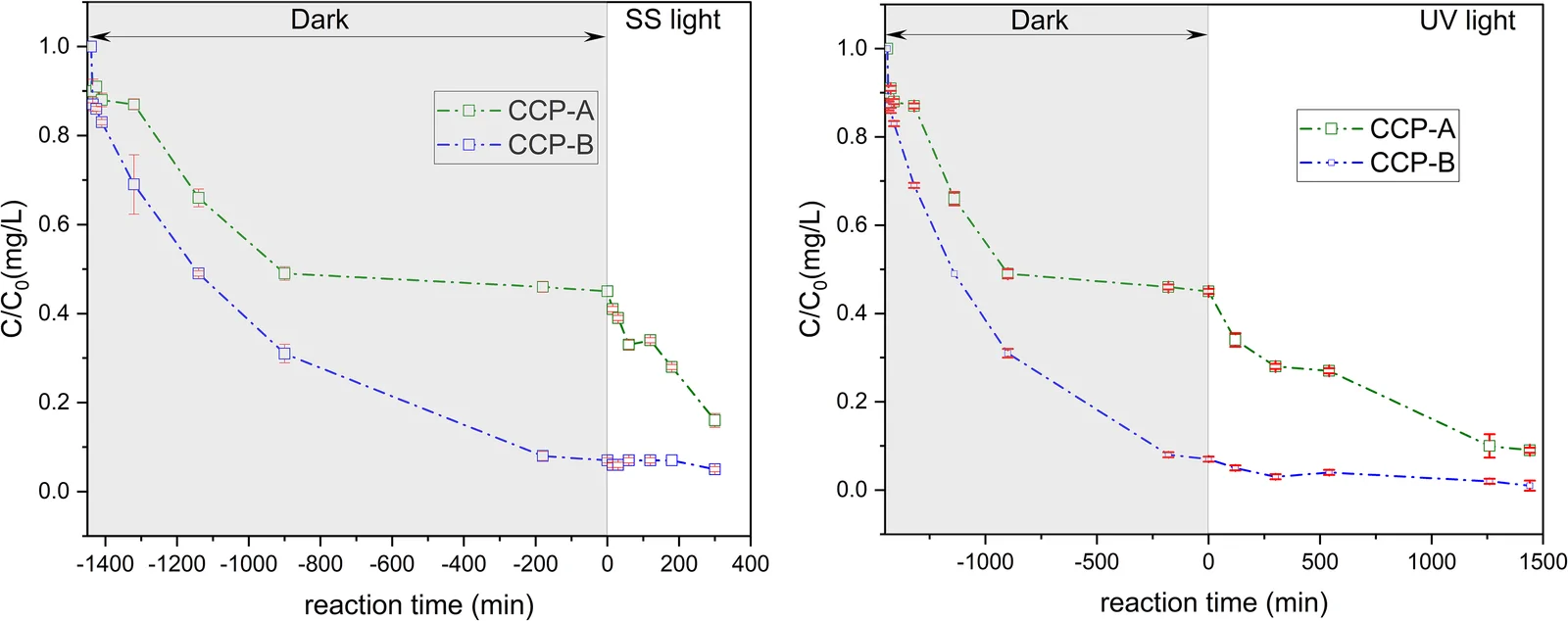
Methylene blue removal in sequential dark adsorption-irradiation experiments. Removal efficiency of CCP-A and CCP-B after 24 h under dark conditions and after the subsequent irradiation stage: 5 h under simulated solar irradiation (left) or 24 h under UV irradiation (right).

Fig 3 compares MB removal by CCP-A and CCP-B in sequential dark adsorption-irradiation experiments. The biochar-MB suspensions were first maintained under dark conditions for 24 h and subsequently exposed either to simulated solar irradiation for 5 h or to UV irradiation for 24 h. Consequently, the values recorded at the end of the dark stage correspond to adsorption after 24 h, whereas the values recorded after irradiation represent the overall outcome of the complete sequential treatment. The final residual MB concentrations obtained after these treatments are reported in Table 3. These results are distinct from those of the independent direct simulated solar irradiation experiment presented in Fig 7, which did not include the preceding 24 h dark adsorption stage.

**Table 3:** Residual methylene blue concentrations after sequential dark adsorption and irradiation treatment. Residual MB concentrations measured after 24 h of contact with CCP-A or CCP-B under dark conditions, followed by either 24 h of UV irradiation or 5 h of simulated solar (SS) irradiation.

Material	24 h dark + 24 h UV (mg/L)	24 h dark + 5 h SS (mg/L)
CCP-A	2.8	4.6
CCP-B	0.4	1.4

From a process-performance standpoint, the comparison between CCP-A and CCP-B provides insight into how metabolite extraction before carbonization affects MB removal during the dark adsorption stage and the subsequent irradiation stage. Under dark conditions, the decrease in MB concentration was attributed to adsorption. Once irradiation was initiated, any additional decrease was reported as overall photo-assisted removal because adsorption and possible light-induced transformations may have occurred concurrently. The corresponding control experiments are presented in S2 Fig in S1 File. Since these processes were not quantified independently, the removal measured after irradiation cannot be attributed exclusively to a light-induced transformation or interpreted as direct evidence of photodegradation.

As shown in Table 3, CCP-B exhibited lower final residual MB concentrations than CCP-A after both sequential treatments: 0.4 versus 2.8 mg/L after 24 h of dark adsorption followed by 24 h of UV irradiation, and 1.4 versus 4.6 mg/L after 24 h of dark adsorption followed by 5 h of simulated solar irradiation. The additional decrease in MB concentration during the irradiation stage is consistent with a contribution from photo-assisted removal beyond that achieved during the preceding dark adsorption stage. However, because adsorption and possible light-induced transformations were not quantified independently during irradiation, their relative contributions cannot be resolved conclusively. The coexistence of adsorption and light-induced transformation has been reported for other carbon-containing systems [1,21]; nevertheless, those studies do not establish that the same mechanisms occurred in CCP-B.

Overall, CCP-B exhibited higher MB removal than CCP-A during both stages of the sequential experiments. After UV irradiation, CCP-B achieved an overall removal efficiency of 98.6%, compared with 90.4% for CCP-A. Its higher removal during the preceding dark stage may be associated with differences in the chemical composition of the materials, including the higher oxygen signal observed by EDS and the oxygen-containing species identified by XPS and FTIR (S9 Fig in S1 File). These features may favor adsorption through electrostatic attraction and hydrogen bonding [20,22]. CCP-B also reached lower residual MB concentrations after irradiation; however, this difference cannot be attributed exclusively to a photochemical mechanism because adsorption and possible light-induced transformations were not separated experimentally.

During the dark adsorption experiments, CCP-B produced a markedly greater decrease in MB concentration than CCP-A, resulting in final residual concentrations of 2.2 and 13.3 mg/L, respectively. Thus, the residual MB concentration obtained with CCP-B was approximately sixfold lower than that obtained with CCP-A. This difference may be associated with the greater overall oxygen content of CCP-B and the oxygen-containing surface species identified by XPS and FTIR. These functionalities may contribute to electrostatic attraction and hydrogen bonding with MB, whereas aromatic carbon domains may support π-π interactions [20,22]. Nevertheless, the available characterization does not quantify the individual contribution or aqueous accessibility of these adsorption sites.

Adsorption of MB by biomass-derived biochar has also been reported for material obtained from *Dacryodes edulis* leaves [23]. Other studies illustrate the importance of different material properties. Li et al. identified a strong relationship between MB adsorption, BET surface area, and micropore volume in activated Starbons® [24], whereas Przytulska et al. reported high MB adsorption by an activated biocarbon prepared from post-extraction residue [25]. These comparisons indicate that adsorption performance depends on the combination of porosity, surface chemistry, and aqueous accessibility rather than on a single material parameter.

**Fig 4:**
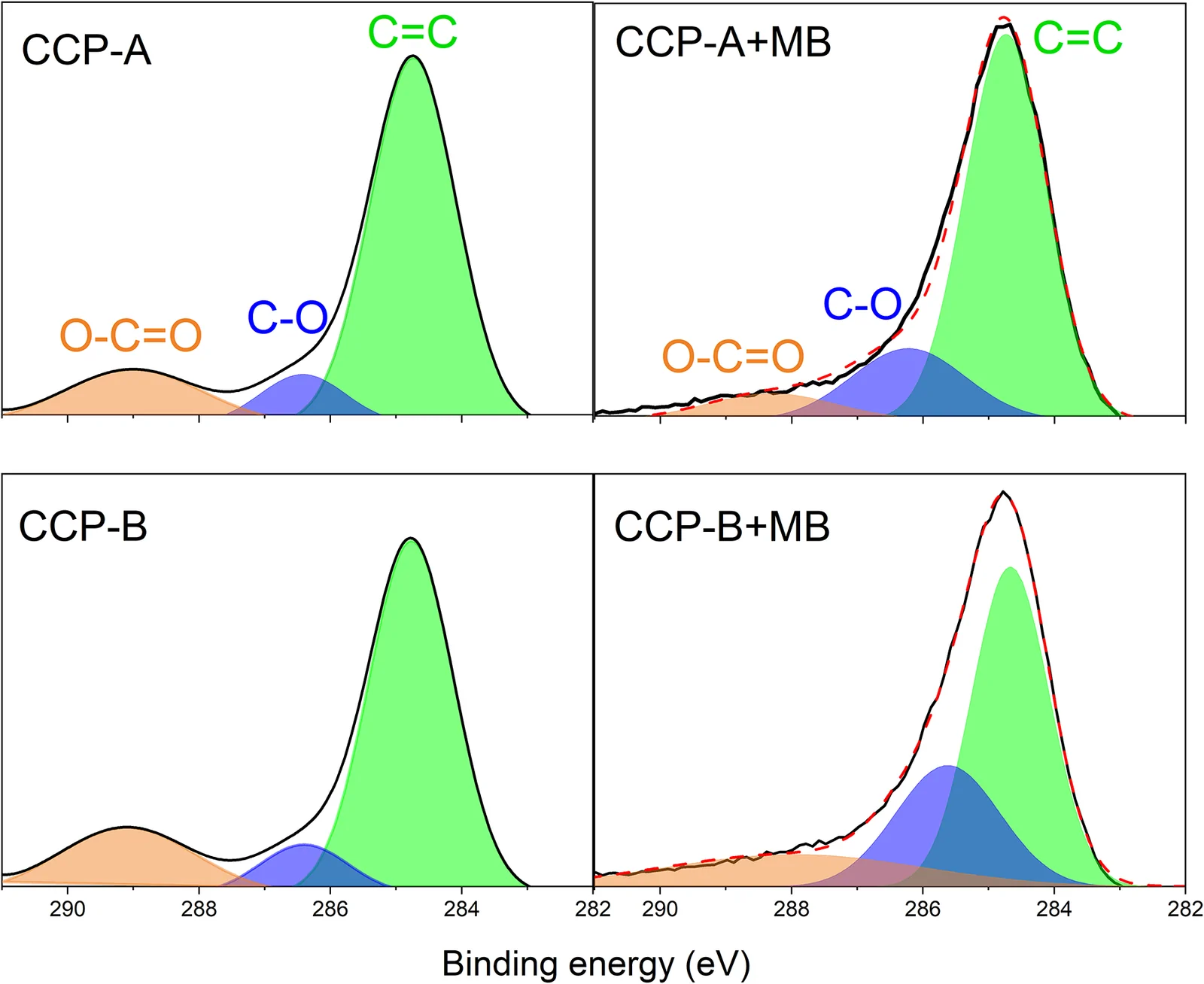
XPS C 1s spectra of chaya-derived carbon materials before and after methylene blue treatment. Spectra of pristine biocarbons (left) and samples recovered after MB treatment under UV irradiation (right), showing changes in carbon bonding environments. Red curves correspond to Gaussian multipeak deconvolution (GaussAmp).

X-ray photoelectron spectroscopy analysis revealed the surface carbon environments of both biocarbons (Fig 4). The C 1s spectra are consistent with oxygen-functionalized carbonaceous materials and complement the structural information obtained by Raman spectroscopy and FTIR. The principal C 1s component at approximately 284.8 eV was assigned to C–C/C = C environments, whereas the components at approximately 286.4 and 289.0 eV were associated with C–O–C and O–C = O or carbonate-related species, respectively. The presence of carbonate-related species is also consistent with the crystalline phases identified by XRD (Fig 1). Aromatic carbon domains may contribute to MB adsorption through π-π interactions between the carbonaceous surface and the aromatic dye [22]. After MB treatment under UV irradiation, decreases in the relative C–C/C = C contribution and increases in the oxygen-containing components were observed (Table 4). The post-treatment spectral changes are consistent with interactions between MB-derived species and the carbonaceous surface [22]. However, XPS alone cannot determine whether these changes result exclusively from adsorption or also involve light-induced transformation.

**Table 4:** XPS surface composition of chaya-derived carbon materials before and after methylene blue treatment. Binding-energy signals and relative contributions for CCP-A and CCP-B before treatment and after MB treatment under UV irradiation.

Signal	Parameter	CCP-A	CCP-A + MB	CCP-B	CCP-B + MB
**C-C/C = C**	X_c_ (eV)	284.70	284.73	284.80	284.87
	RP (%)	77.72	63.21	75.23	53.86
**C-O-C**	X_c_ (eV)	286.40	286.52	286.40	285.68
	RP (%)	10.62	18.84	10.57	23.79
**O-C = O**	X_c_ (eV)	289.00	289.12	289.05	289.25
	RP (%)	11.66	17.93	14.20	22.35

**Fig 5:**
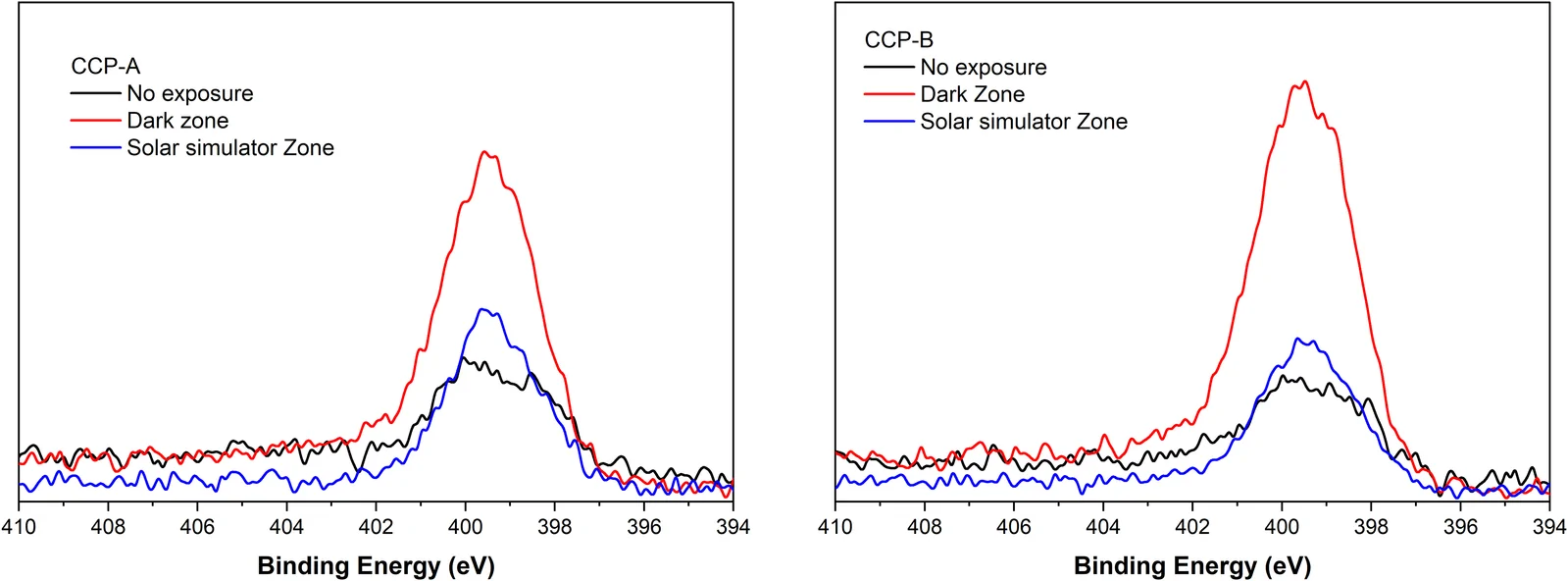
XPS N 1s spectra of chaya-derived carbon materials under different conditions. Spectra of pristine samples, after MB treatment under dark conditions and after MB treatment under irradiation.

Changes in the relative proportions of the C 1s components were observed after MB treatment. The decrease in the relative C–C/C = C contribution and the corresponding increases in C–O–C and O–C = O components are consistent with changes in surface composition following contact with MB [20]. These results support the possible involvement of oxygen-containing functionalities in dye–surface interactions but do not demonstrate that these groups dominate the adsorption mechanism. Likewise, the N 1s signal indicates retention of nitrogen-containing MB-derived species on the materials (Fig 5). The stronger N signal observed for CCP-B after dark exposure is consistent with its greater adsorption under dark conditions, whereas the lower signal following irradiated treatment may reflect a combination of adsorption, desorption, and possible light-induced transformation. These XPS results do not establish that a photochemical mechanism predominates under illumination.

### Kinetic and equilibrium modeling of MB adsorption on CCP-B

To compare the adsorption kinetics of MB on CCP-B, the experimental data were fitted using four commonly applied models: pseudo-first-order, pseudo-second-order, intraparticle-diffusion, and Elovich. The fitted curves are shown in S4 Fig in S1 File, and the corresponding parameters, R^2^ values, and adjusted RMSE values are summarized in Table 5.

**Table 5:** Kinetic parameters for methylene blue adsorption on CCP-B. Model constants and fitting parameters obtained using the pseudo-first-order, pseudo-second-order, intraparticle-diffusion, and Elovich models.

	Pseudo-first order	Pseudo-second order	Intra-particle diffusion	Elovich
R^2^	0.9632	0.9720	0.9122	0.8887
Adjusted RMSE (mg g-1)	10.58	9.22	16.33	18.39
Constants	k_1_ = 0.0029 min^-1^	k_2_ = 1.97 x 10^-5^ g mg^-1^ min^-1^	k_i_ = 19.7697 mg/(g·min^½^)	β= 0.0388
	q_e_ = 135.54 mg/g	q_e_ = 163.28 mg/g	--	α= 1.0903

Among the evaluated models, the pseudo-second-order model provided the highest $R^{\text{2}}$ (0.9720) and the lowest adjusted RMSE (9.22 mg g^-1^), whereas the pseudo-first-order model showed a closely comparable fit ($R^{\text{2}}=\text{0}.\text{9}\text{6}\text{3}\text{2}$; adjusted RMSE = 10.58 mg g^-1^). However, both models overestimated the experimental equilibrium adsorption capacity of approximately 134 mg g^-1^, yielding predicted values of 135.54 and 163.28 mg g^-1^, respectively. The intraparticle-diffusion model showed lower agreement with the experimental data ($R^{\text{2}}=\text{0}.\text{9}\text{1}\text{2}\text{2}$; adjusted RMSE = 16.33 mg g^-1^), indicating that this model described the kinetic data less accurately than the pseudo-order models. The Elovich model provided the weakest fit ($R^{\text{2}}=\text{0}.\text{8}\text{8}\text{8}\text{7}$; adjusted RMSE = 18.39 mg g^-1^). Model comparison was therefore based on both $R^{\text{2}}$ and adjusted RMSE rather than on $R^{\text{2}}$ alone [26].

Overall, the kinetic-model comparison was consistent with multiple transport contributions, but it did not establish a unique rate-controlling step or a specific molecular interaction mechanism. These results describe adsorption under dark conditions and should be interpreted separately from the removal efficiencies measured under irradiation, which represent overall photo-assisted removal.

Equilibrium data were fitted using the Langmuir, Freundlich, and Temkin models (S5 Fig in S1 File). The Langmuir model yielded the highest linearized coefficient of determination ($R^{\text{2}}=\text{0}.\text{9}\text{7}$; adjusted RMSE = 13.93 mg g^-1^), whereas the Freundlich ($R^{\text{2}}=\text{0}.\text{7}\text{4}$; adjusted RMSE = 13.40 mg g^-1^) and Temkin models ($R^{\text{2}}=\text{0}.\text{8}\text{4}$; adjusted RMSE = 13.17 mg g^-1^) showed slightly lower adjusted RMSE values on the original $q_{e}$ scale. Thus, although Langmuir provided the highest $R^{\text{2}}$, the complementary adjusted-RMSE analysis did not identify it unequivocally as the best-fitting model.

The Langmuir fit yielded a model-estimated q_max_ of 155.3 mg g^-1^ and an equilibrium constant, b, of 2.64 L mg^-1^, whereas the Freundlich exponent, n=3.08, indicated favorable adsorption within the tested concentration range.

Because q_max_ was obtained by model fitting rather than measured directly at complete saturation, the Langmuir fit should not be interpreted as conclusive evidence of monolayer adsorption on a homogeneous surface. The comparatively high model-estimated q_max_ and low N_2_-BET surface area of CCP-B (6.0 m^2^ g^-1^) indicate that MB uptake cannot be described solely by the dry surface area accessible to N_2_ at 77 K. This behavior falls within the broad variability reported for biomass-derived adsorbents. For example, Nnaji et al. reported an equilibrium uptake of up to 28.94 mg g^-1^ for carbonized *Dacryodes edulis* leaves with a specific surface area of 0.983 m^2^ g^-1^, whereas Liu et al. reported adsorption capacities ranging from 99.9 to 251.08 mg g^-1^ for corn-straw biochars, with differences associated with precursor-dependent surface characteristics. By contrast, an activated biocarbon prepared from post-extraction horsetail residue exhibited a substantially larger BET surface area of 964 m^2^ g^-1^ and a Langmuir q_max_ of 334.4 mg g^-1^ [20,23,25]. These values are not directly comparable because precursor composition, activation, solution pH, adsorbent dosage, concentration range, and fitting procedures differ; nevertheless, they illustrate that MB adsorption capacity does not scale uniformly with N_2_-BET surface area across biomass-derived carbonaceous materials. For CCP-B, oxygen-containing functionalities identified by FTIR and XPS may contribute to electrostatic attraction and hydrogen bonding, whereas aromatic carbon domains may support π-π interactions with MB [20,22]. Hydration or swelling may also modify site accessibility under aqueous conditions. However, the present characterization does not quantify aqueous site accessibility or the individual contribution of these interactions. Additional zeta-potential measurements, aqueous-phase porosity analysis, and direct experimental verification of the saturation capacity would therefore be required to explain this behavior conclusively.

### Performance of materials after multiple use cycles

**Fig 6:**
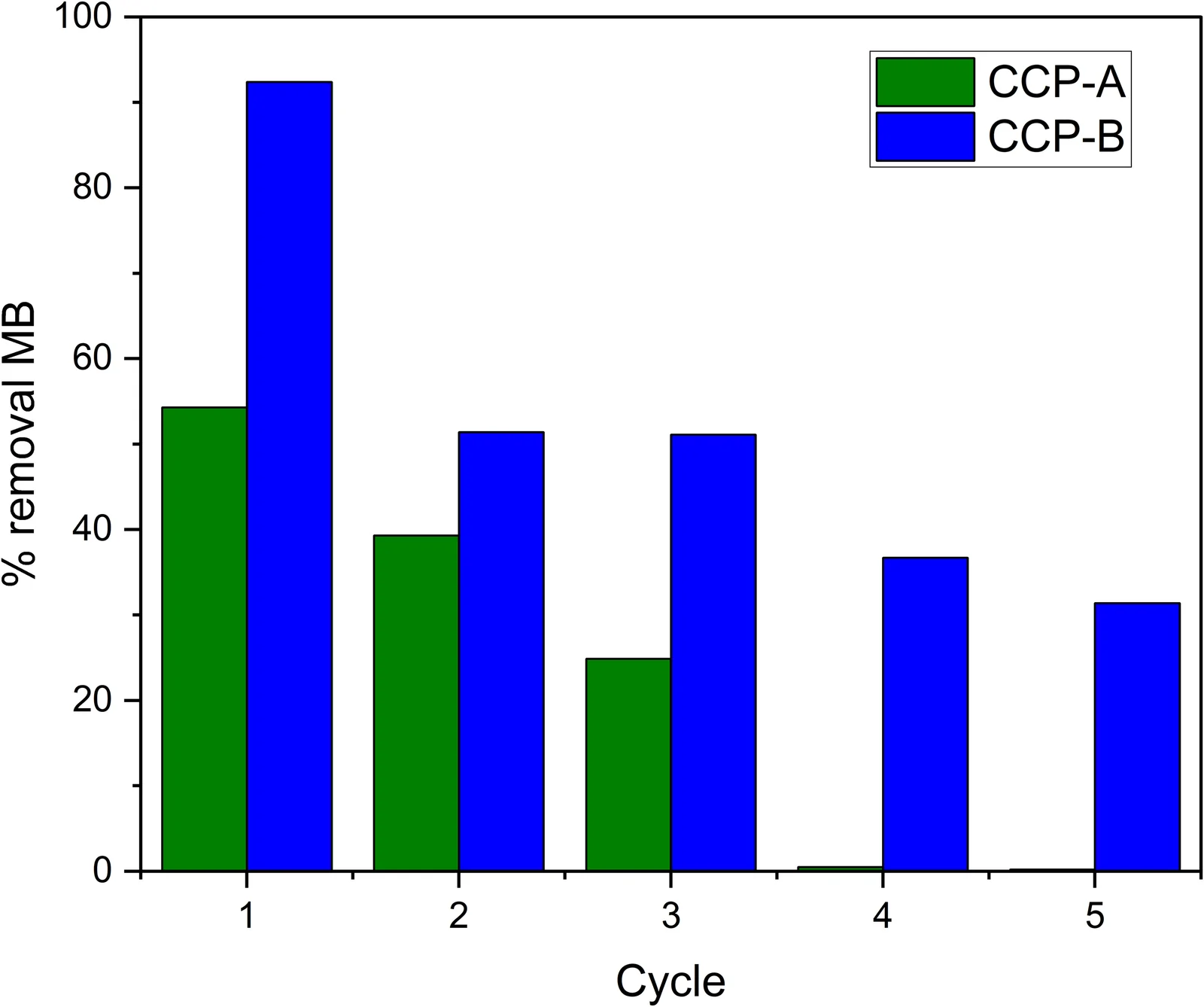
Methylene blue removal efficiency over repeated adsorption-drying cycles. Removal efficiency of CCP-A and CCP-B over five consecutive reuse cycles in which the recovered MB-loaded materials were vacuum-dried without a chemical, solvent-based, or thermal desorption treatment.

To assess short-term reuse performance under a minimal inter-cycle treatment, CCP-A and CCP-B were evaluated over five consecutive adsorption cycles. After each cycle, the MB-loaded adsorbent was separated, vacuum-dried for 8 h, and re-exposed to a fresh MB solution under identical experimental conditions. Because no chemical, solvent-based, or thermal desorption step was performed, this procedure represents consecutive adsorption-drying reuse cycles rather than adsorption-desorption or fully regenerated cycles. As shown in Fig 6, the removal efficiency of both materials decreased with successive reuse, although CCP-B maintained higher removal efficiencies than CCP-A throughout the experiment.

CCP-B removed approximately 92% of MB in the first cycle, 51% in the second and third cycles, 36% in the fourth cycle, and 31% in the fifth cycle. Thus, by the fifth cycle, its removal efficiency corresponded to only approximately 34% of its initial value. CCP-A showed an even sharper decline, falling below 30% removal by the third cycle. Although CCP-B consistently outperformed CCP-A, the pronounced loss of performance, particularly between the first and second cycles, indicates limited practical reusability under the inter-cycle treatment employed here. Vacuum drying removes water but does not deliberately desorb the retained MB; therefore, persistent occupation of adsorption sites and pore blocking may have contributed to the decrease. However, material loss during recovery, leaching of mineral or surface components, and structural or chemical alteration of the biochars cannot be excluded.

The present experiment evaluates the retention of removal performance but does not establish the structural or chemical stability of the materials because adsorbent mass recovery, component leaching, desorption efficiency, and post-cycle physicochemical properties were not determined. Consequently, the current results are insufficient to support direct repeated application without an effective regeneration procedure. Further studies should optimize and compare regeneration strategies, quantify adsorbent recovery and desorption efficiency, characterize the materials after repeated cycles, and assess the energy demand, chemical consumption, and secondary waste associated with regeneration before practical wastewater-treatment applications can be considered.

### Behavior under direct simulated solar irradiation

**Fig 7:**
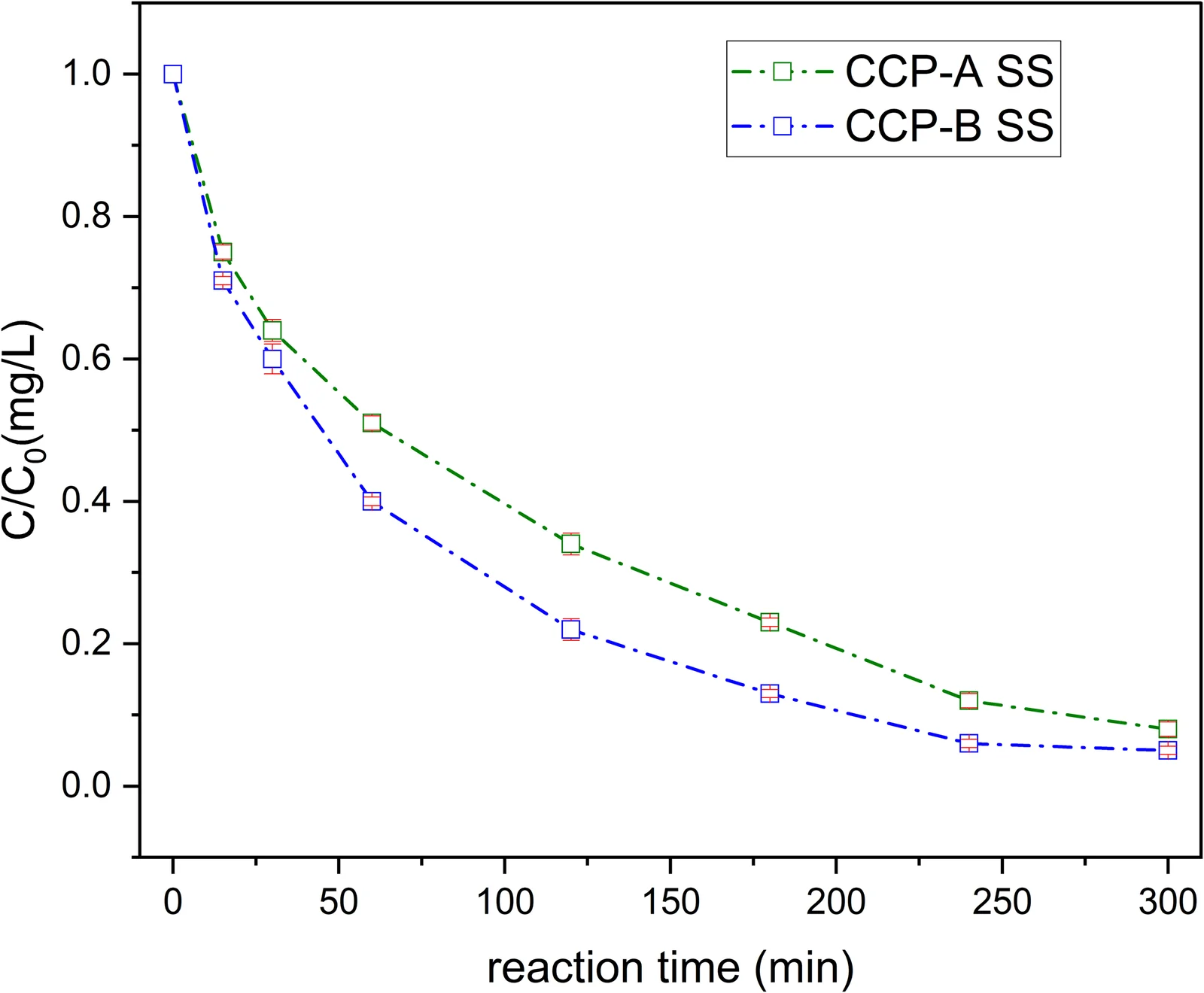
Methylene blue removal kinetics during direct simulated solar irradiation. Changes in residual MB concentration during 5 h of simulated solar irradiation in the presence of CCP-A and CCP-B. This experiment was conducted independently and did not include the preceding 24 h dark adsorption stage used for the results presented in Fig 3 and Table 3.

In a separate experiment, MB concentration was monitored during 5 h of direct simulated solar irradiation in the presence of CCP-A or CCP-B, without the preceding 24 h dark adsorption stage used in the sequential experiments presented in Fig 3 and Table 3. The residual MB concentrations after 5 h were 2.61 mg/L for CCP-A and 1.79 mg/L for CCP-B (Fig 7). Both materials exhibited a gradual decrease in MB concentration during irradiation, with CCP-B reaching the lower final concentration. These results describe the overall removal behavior under direct simulated solar irradiation. Since adsorption and possible light-induced transformations were not quantified independently, the observed decrease cannot be attributed exclusively to a photochemical process.

To examine spectral changes associated with adsorption and photo-assisted removal, full UV-Vis spectra in the 200–800 nm range were recorded and analyzed by Gaussian deconvolution (S7 Fig in S1 File). The reference MB spectrum showed a principal band at 664 nm, associated with the monomeric chromophore, and a shoulder at approximately 610–620 nm, associated with dimeric or aggregated species. After contact with the carbonaceous materials, decreases in absorbance intensity, band broadening, and small spectral shifts were observed. These changes may reflect dye-surface interactions, changes in MB aggregation, and possible light-induced transformation. However, UV-Vis spectral changes alone cannot distinguish unequivocally among these processes. Therefore, the similarity of the spectra obtained under irradiation does not demonstrate that photochemical mechanisms predominate over adsorption. The results provide evidence of changes in the MB spectral profile but do not constitute direct proof of molecular degradation.

**Fig 8:**
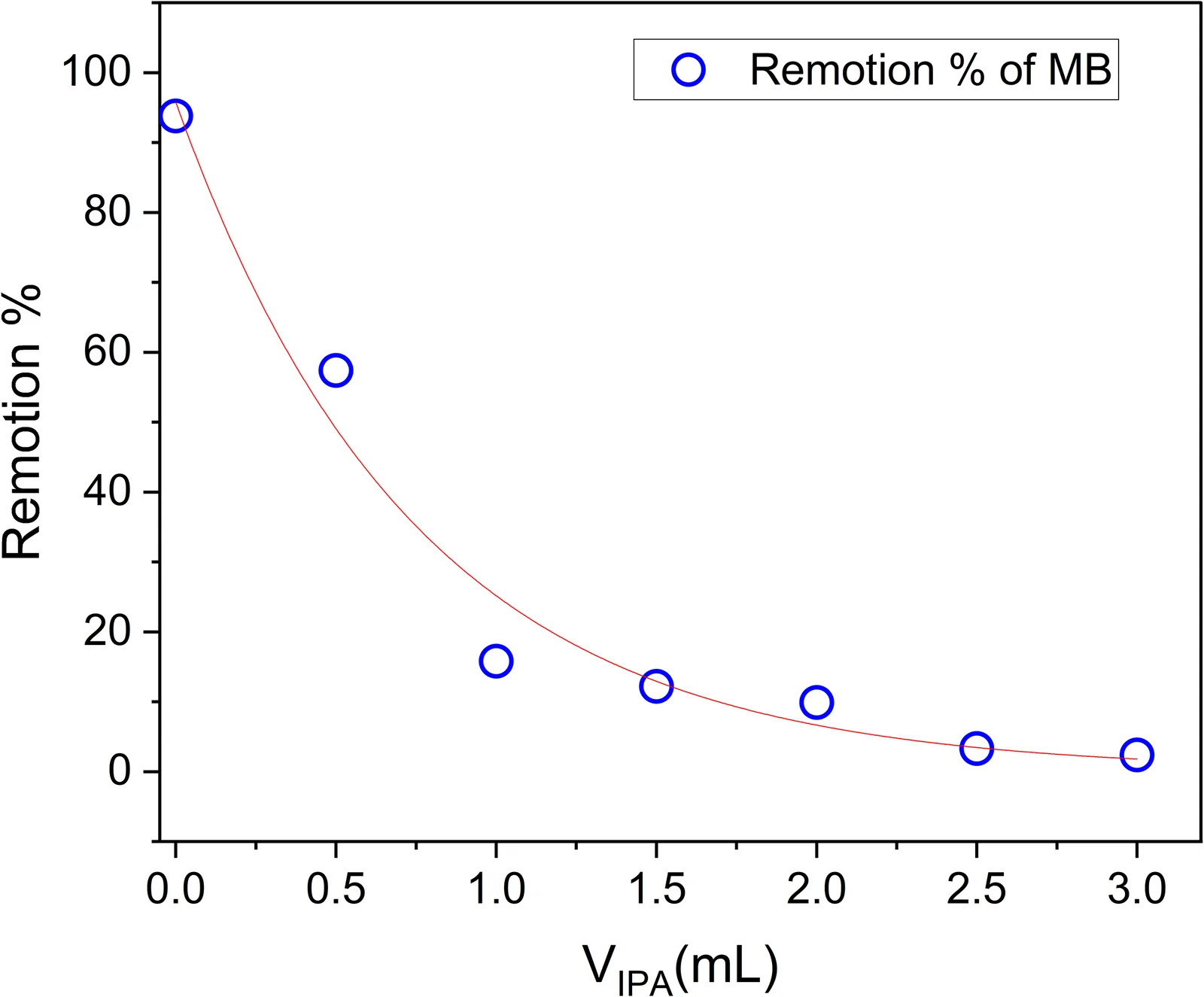
Effect of isopropanol (IPA) on methylene blue removal. Removal efficiency (%) as a function of the volume of IPA added, used as a hydroxyl radical scavenger.

To examine the possible participation of reactive species in photo-assisted removal, scavenging experiments were performed using isopropanol (IPA), a commonly used scavenger of hydroxyl radicals (•OH). The results are summarized in Fig 8. In the absence of IPA, CCP-B achieved 93.8% MB removal after 5 h under simulated solar irradiation. However, increasing the volume of IPA caused a pronounced decline in removal efficiency, from 57.4% with 0.5 mL of IPA to below 3% with 2.5–3.0 mL.

This decrease is consistent with the possible participation of hydroxyl radicals or related reactive oxygen species in photo-assisted removal. However, the evidence is indirect because IPA may also affect dye-surface interactions and the solution environment. Direct ROS detection, mineralization measurements, and degradation-product identification would be required to identify the reactive species involved and establish an oxidation pathway.

**Fig 9:**
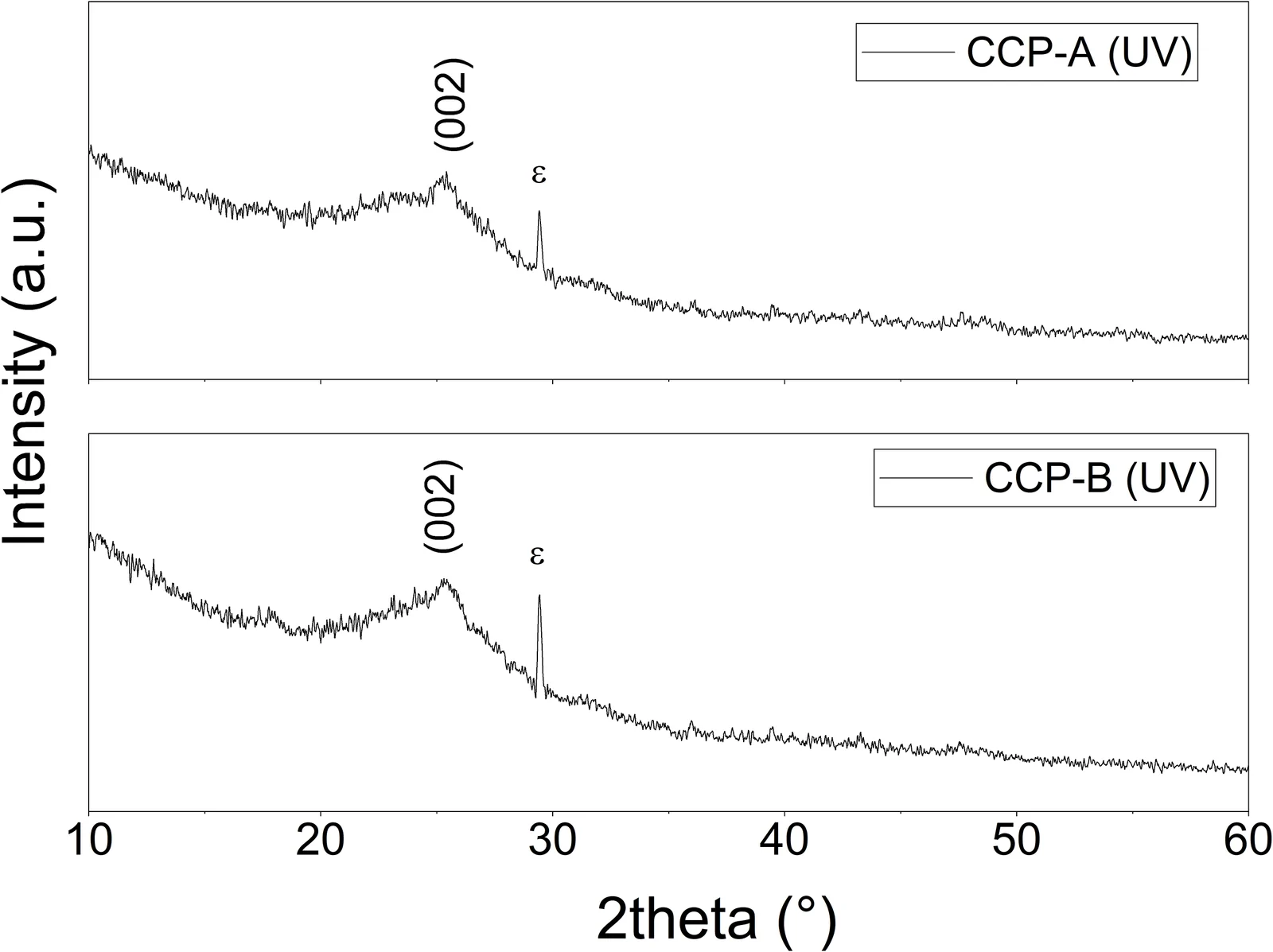
XRD patterns of chaya-derived carbon materials after UV irradiation during methylene blue treatment. X-ray diffraction patterns of CCP-A and CCP-B after UV exposure, showing the persistence of crystalline CaCO_3_ (ε phase).

XRD patterns recorded after UV-assisted MB treatment showed lower CaCO_3_ peak intensities, while the broad carbon (002) feature at approximately 25° persisted (Fig 9). The mineral-phase change and the increase in final pH (Table 6) are consistent with dissolution and acid-base equilibria involving alkaline mineral components and surface functional groups [27–30]. A possible calcium-containing equilibrium pathway is schematically presented in S6 Fig in S1 File; however, it does not constitute direct evidence of Ca(OH)_2_ formation. These changes may alter adsorption-site accessibility, but neither XRD nor pH measurements demonstrate enhanced adsorption or MB degradation. Consequently, contributions from aromatic carbon domains or reduced obstruction by surface mineral phases remain mechanistic hypotheses [31,32].

**Table 6:** pH values of methylene blue solutions before and after treatment. pH values measured before and after treatment with CCP-A and CCP-B.

Material	Initial pH	Final pH
CCP-A	4.80	7.44
CCP-B	4.85	9.01

**Fig 10:**
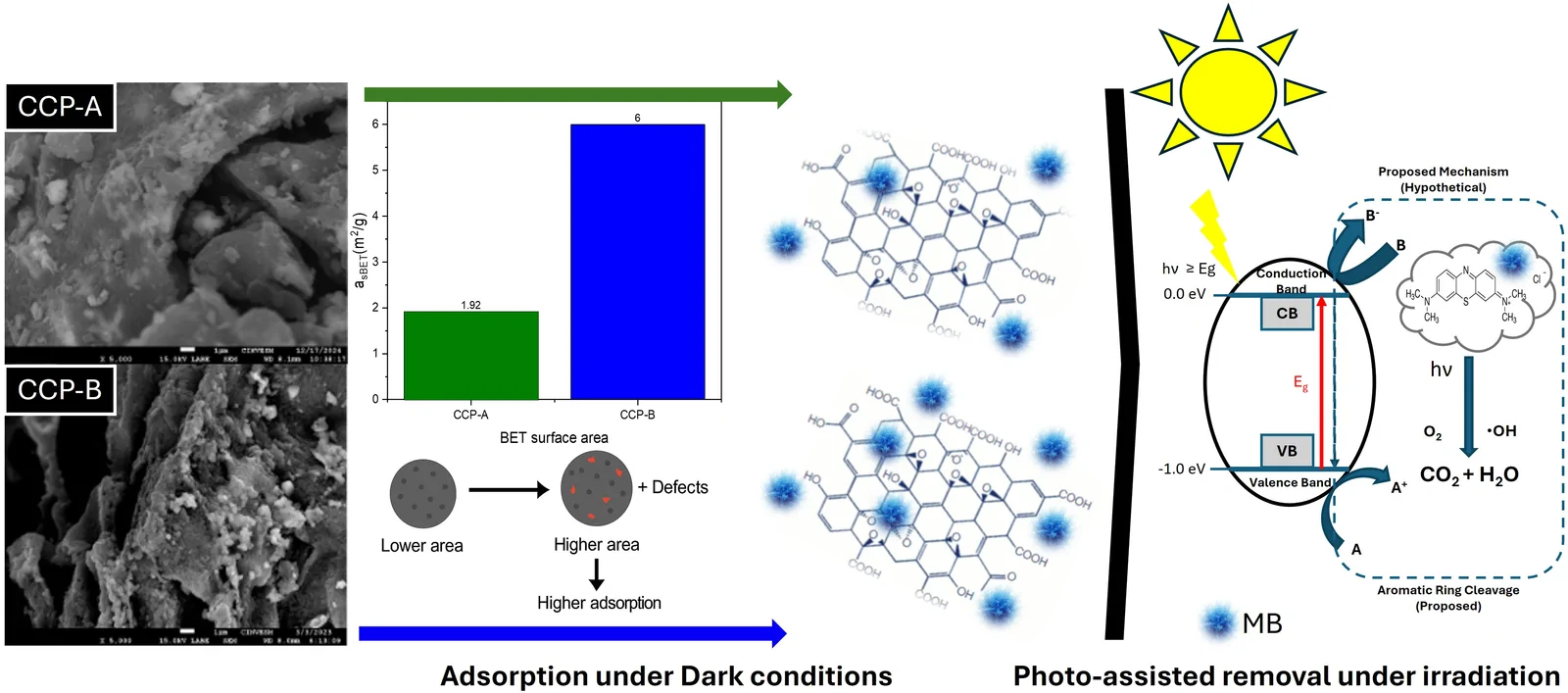
Operational framework proposed for methylene blue removal using chaya-derived carbon materials. The scheme distinguishes adsorption under dark conditions from overall photo-assisted removal under irradiation. Possible light-induced transformation processes and reactive oxygen species participation are presented as hypotheses rather than experimentally demonstrated pathways.

Taken together, the results support the following operational framework for MB removal (Fig 10), although they do not provide direct evidence of photodegradation or mineralization:

- Adsorption under dark conditions. MB uptake may involve π-π interactions with aromatic carbon domains, electrostatic attraction, and hydrogen bonding involving oxygen-containing surface groups.
- Photo-assisted removal under irradiation. Overall MB removal reflects adsorption occurring concurrently with possible light-induced transformation processes. Radical scavenging results are consistent with a possible contribution from hydroxyl radicals or related reactive oxygen species, but this contribution remains indirect.

## Conclusions

This study shows that, under the conditions evaluated, metabolite extraction prior to carbonization modified the physicochemical properties of chaya-derived biochar and influenced its performance for methylene blue removal from water. Compared with the biochar produced from untreated biomass (CCP-A), the post-extraction material (CCP-B) showed greater MB adsorption under dark conditions and higher overall removal under UV and simulated solar irradiation, reaching removal efficiencies of 98.6% and 93.8%, respectively. The improved performance of CCP-B cannot be attributed exclusively to either its BET surface area or its surface chemistry. XPS and FTIR analyses confirmed the presence of oxygen-containing functionalities that may contribute to electrostatic attraction and hydrogen bonding, whereas aromatic carbon domains may support π-π interactions with MB. Differences in pore structure and the accessibility of adsorption sites in aqueous media may also contribute; however, the relative importance of these properties could not be determined from the available characterization.

Kinetic analysis was consistent with multiple transport contributions, although a unique rate-controlling step could not be assigned. Among the equilibrium models, Langmuir yielded the highest linearized R^2^, whereas degrees-of-freedom-adjusted RMSE values on the original q_e_ scale were slightly lower for the Temkin and Freundlich models. Therefore, no single equilibrium model was unequivocally superior, and the Langmuir fit should not be interpreted as conclusive evidence of monolayer adsorption on a homogeneous surface. Under irradiation, the observed decrease in MB concentration is described as photo-assisted removal because adsorption and possible light-induced transformations may occur concurrently. Changes in the UV-Vis spectra may reflect dye-surface interactions, changes in MB aggregation, or possible light-induced transformations, whereas inhibition by isopropanol is consistent with the possible participation of hydroxyl radicals or related reactive oxygen species. Nevertheless, these observations constitute indirect evidence and do not demonstrate a specific photodegradation pathway, complete degradation, or mineralization. Direct detection of reactive oxygen species, identification of transformation products, and mineralization measurements would be required to establish the underlying mechanism conclusively.

CCP-B was prepared without chemical activation and consistently outperformed CCP-A. Nevertheless, its removal efficiency decreased from approximately 92% in the first cycle to 31% in the fifth cycle under the vacuum-drying-only inter-cycle treatment, demonstrating that performance was not maintained during repeated use. Because no effective desorption procedure, adsorbent mass recovery analysis, leaching assessment, or post-cycle physicochemical characterization was performed, long-term material stability and practical reusability remain unestablished. Overall, the paired comparison shows that, for the chaya-isopropanol-MB system evaluated, metabolite extraction before carbonization was associated with changes in biochar properties and improved MB-removal performance. However, additional extraction procedures, thermal-treatment conditions, biomass sources, pollutants, and realistic water matrices must be evaluated before the practical relevance and broader applicability of this approach can be established.

## Supporting information

S1 File Supporting information file containing supplementary Figs S1-S10, including UV-Vis spectra and calibration data, irradiation controls, N_2_ adsorption-desorption isotherms and textural parameters, kinetic and equilibrium model fits, proposed calcium-containing equilibria, spectral deconvolution, SEM-EDS analyses, ATR-FTIR spectra, and TGA/DTG profiles. (DOCX)
