# Supplementary material for: Methylene blue removal from water using chaya-derived biochar: Effect of metabolite extraction on adsorption
Source: PLoS One. 2026 Sep 24;21(9):e0358748. doi: 10.1371/journal.pone.0358748 (PMC13607801; doi:10.1371/journal.pone.0358748)
Supplement: Supplementary file 1 [file pone.0358748.s001.docx]

Supporting Information file

**Methylene blue removal from water using chaya-derived biochar: Effect of metabolite extraction on adsorption**

Viviana Roche-Llerena^1♦^, Leonardo Hernández^1♦^, Raúl Pareja-Rodríguez^1^, Geonel Rodríguez-Gattorno^1^*, and María A. Fernández-Herrera^1^*

^1^ Departamento de Física Aplicada, Centro de Investigación y de Estudios Avanzados del Instituto Politécnico Nacional, Unidad Mérida. Km 6 Antigua Carretera a Progreso. Apdo. Postal 73, Cordemex, 97310, Mérida, Yuc., México.

^♦^ These authors contributed equally to this work.

Corresponding author: María A. Fernández-Herrera [mfernandez@cinvestav.mx](mailto:mfernandez@cinvestav.mx)

Corresponding author: Geonel Rodríguez-Gattorno [geonelr@cinvestav.mx](mailto:geonelr@cinvestav.mx)

**S1 Fig. UV-Vis spectra and calibration curve for methylene blue quantification.** (a) Absorption spectra of MB standards from 2.5 to 30 mg L^-1^. (b) Calibration curve at 664 nm used to determine MB concentration. The fitted relationship was $A=0.08012C+0.0608$, where $A$ is absorbance and $C$ is MB concentration in mg L^-1^.

**S2 Fig. UV-Vis spectra of methylene blue under the irradiation conditions used in this study.** Spectra of the MB control irradiated without carbonaceous material (reference) and of the residual solutions obtained in the presence of CCP-A or CCP-B after UV irradiation for 24 h (left) or simulated solar irradiation for 5 h (right).

.

**S3 Fig. N_2_ adsorption-desorption isotherms and textural parameters of chaya-derived carbon materials.** Isotherms of CCP-A (left) and CCP-B (right). The table summarizes the BET surface area ($S_{\mathrm{BET}}$), total pore volume ($V_{p}$), and mean pore diameter ($d_{p}$).

| **Sample** | **S_BET_ (m^2^ g^-1^)** | **V_p_ (cm^3^ g^-1^)** | **d_p_ (nm)** |
| --- | --- | --- | --- |
| **CCP-A** | 1.92 | 6.94 ·10^-3^ | 14.13 |
| **CCP-B** | 6.10 | 1.39 ·10^-2^ | 8.42 |

**S4 Fig. Kinetic model fits for methylene blue adsorption on CCP-B.** Experimental data and fits to the (a) pseudo-first-order, (b) pseudo-second-order, (c) intraparticle-diffusion, and (d) Elovich models.

**S5 Fig. Linearized isotherm models for methylene blue adsorption on CCP-B.** (a) Langmuir ($C_{e}/q_{e}$ versus $C_{e}$), (b) Freundlich ($\ln q_{e}$ versus $\ln C_{e}$), and (c) Temkin ($q_{e}$ versus $\ln C_{e}$) fits.

**S6 Fig. Proposed calcium-containing equilibria during methylene blue removal.** Schematic representation of CaCO_3_ dissolution and subsequent acid-base equilibria involving Ca^2+^, carbonate species, OH^-^, and Ca(OH)_2_. The scheme represents a possible pathway and does not constitute experimental evidence of Ca(OH)_2_ formation.

**S7 Fig. UV-Vis spectra and Gaussian deconvolution of methylene blue before and after the sequential irradiation treatments.** (a) Reference MB spectrum before treatment. (b) Spectra obtained after treatment with CCP-A or CCP-B under UV irradiation (top) or simulated solar irradiation (bottom). The colored bands represent the fitted spectral components, and the blue curves represent the cumulative fits.

a)

b)

**S8 Fig. SEM micrographs and EDS elemental maps of chaya-derived carbon materials before and after methylene blue exposure.** SEM images of CCP-A and CCP-B before exposure and after MB treatment. The post-exposure EDS maps show the spatial distributions of C, O, N, and S. Scale bars and magnifications are indicated in the images.

| CCP-A before exposure | |
| --- | --- |
|  |  |
|  |  |

**S8 Fig. (continued)**

| CCP-B before exposure | |
| --- | --- |
|  |  |
|  |  |

**S8 Fig. (continued)**

| CCP-A after exposure | |
| --- | --- |
|  |  |
|  | |

**S8 Fig. (continued)**

| CCP-B after exposure | |
| --- | --- |
|  |  |
|  | |

**S9 Fig. ATR–FTIR spectra of chaya-derived carbon materials.** Spectra of CCP-A (left) and CCP-B (right) recorded over the range of 4000-600 cm^-1^.

**S10 Fig. Thermogravimetric and derivative thermogravimetric profiles of chaya precursors.** TGA and DTG curves recorded under flowing air (25 mL min^-1^) at a heating rate of 10 °C min^-1^ for (a) dehydrated chaya leaves from ambient temperature to 600 °C and (b) the post-extraction residue from ambient temperature to 800 °C.
